# Supplementary material for: Physical Rehabilitation Needs in the BRICS Nations from 1990 to 2017: Cross-National Analyses Using Data from the Global Burden of Disease Study
Source: Int J Environ Res Public Health. 2020 Jun 10;17(11):4139. doi: 10.3390/ijerph17114139 (PMC7312462; doi:10.3390/ijerph17114139)
Supplement: Supplementary file 1 [file ijerph-17-04139-s001.pdf]

Supplementary Material 1: Total Physical Rehabilitation Needs

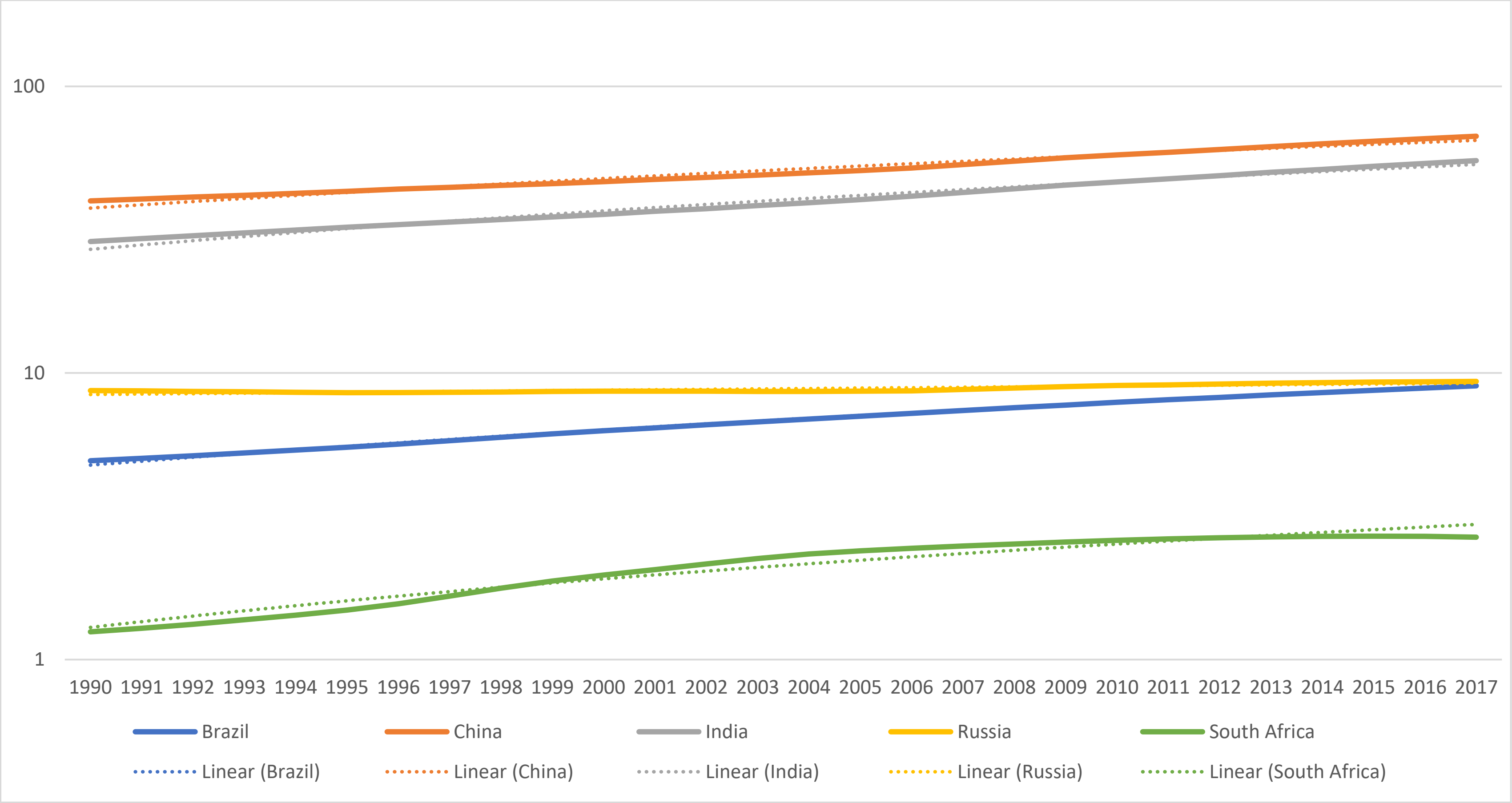

Supplementary Figure 1: Years Lived with Disability (YLD Counts) likely benefiting from physical rehabilitation across the five countries analyzed [1990-2017] and their *best fit* regression models (dotted lines). Values in millions. *Y* axis is in a logarithmic scale.

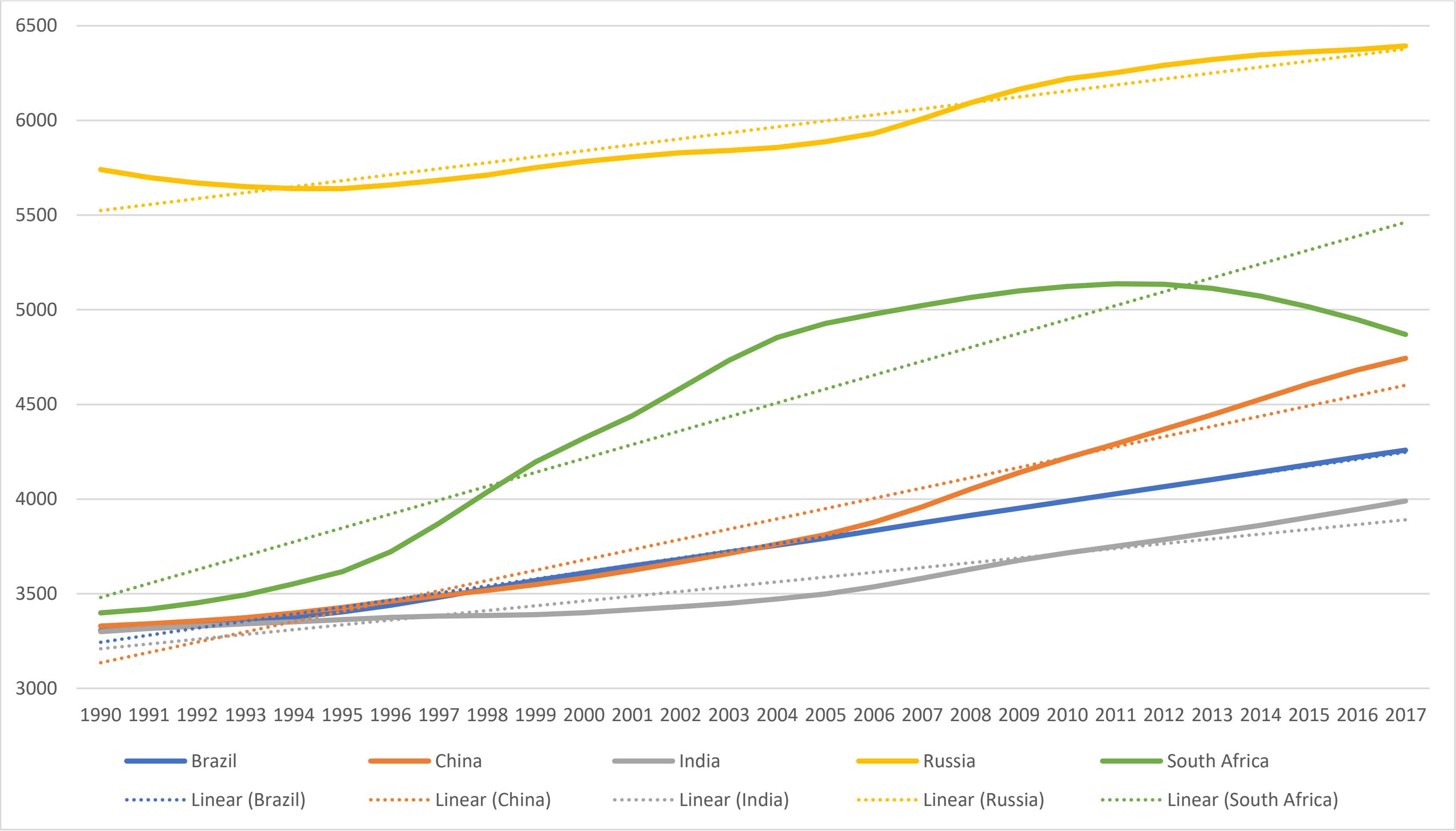

Supplementary Figure 2: YLD Rates (i.e. Years Lived with Disability per 100,000 inhabitants) likely benefiting from physical rehabilitation across the five countries analyzed [1990-2017] and their *best fit* regression models (dotted lines).

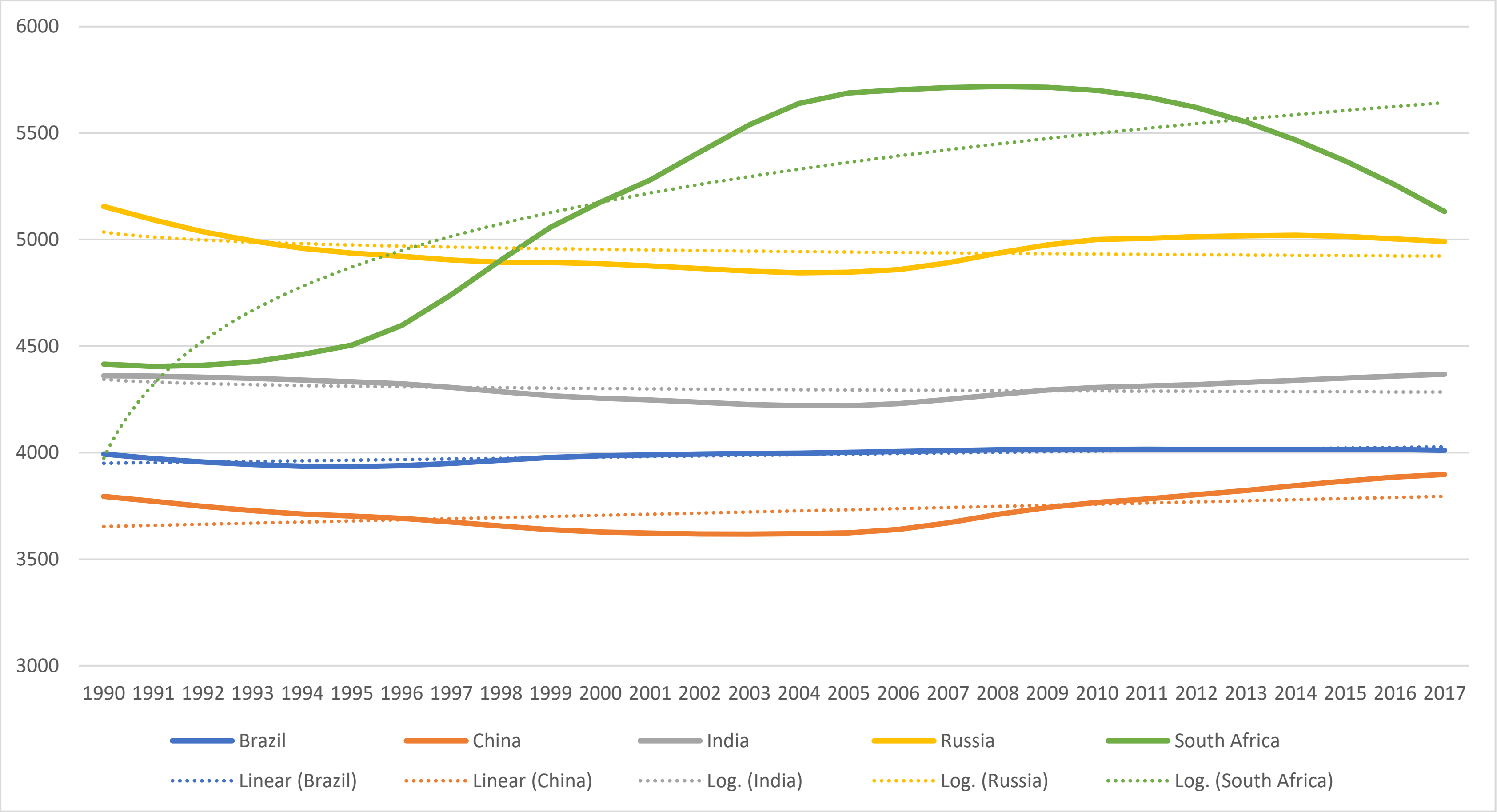

Supplementary Figure 3: Age-standardized YLD Rates (i.e. Years Lived with Disability per 100,000 inhabitants adjusted for population ageing) likely benefiting from physical rehabilitation across the five countries analyzed [1990-2017] and their *best fit* regression models (dotted lines). Abbreviation: Log.: Logarithmic.

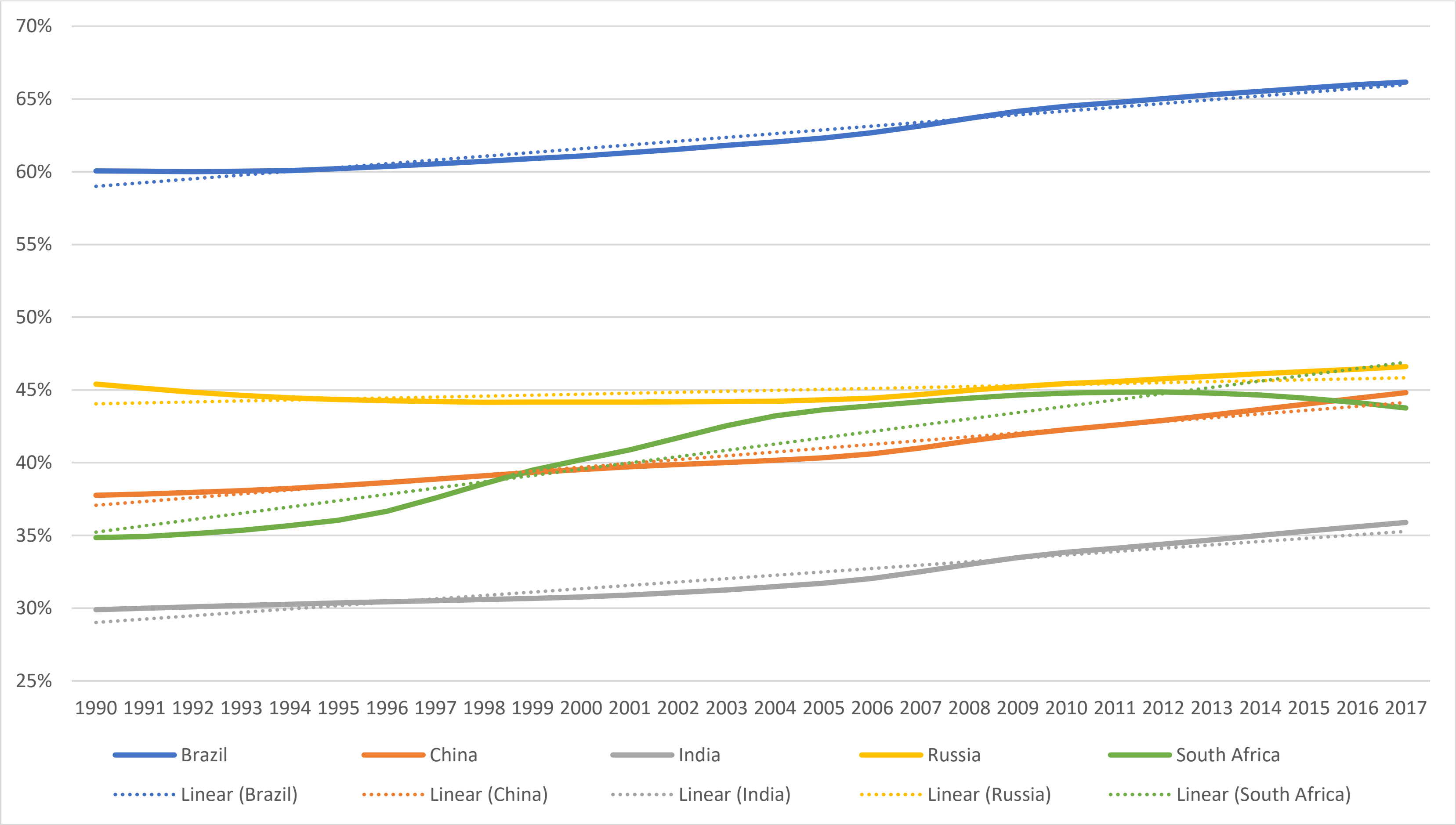

Supplementary Figure 4: Percentage of Years Lived with Disability (YLDs) for all conditions likely benefiting from physical rehabilitation among total YLDs, across the five countries analyzed [1990-2017] and their *best fit* regression models (dotted lines).

Supplementary Material 2: Needs by condition type

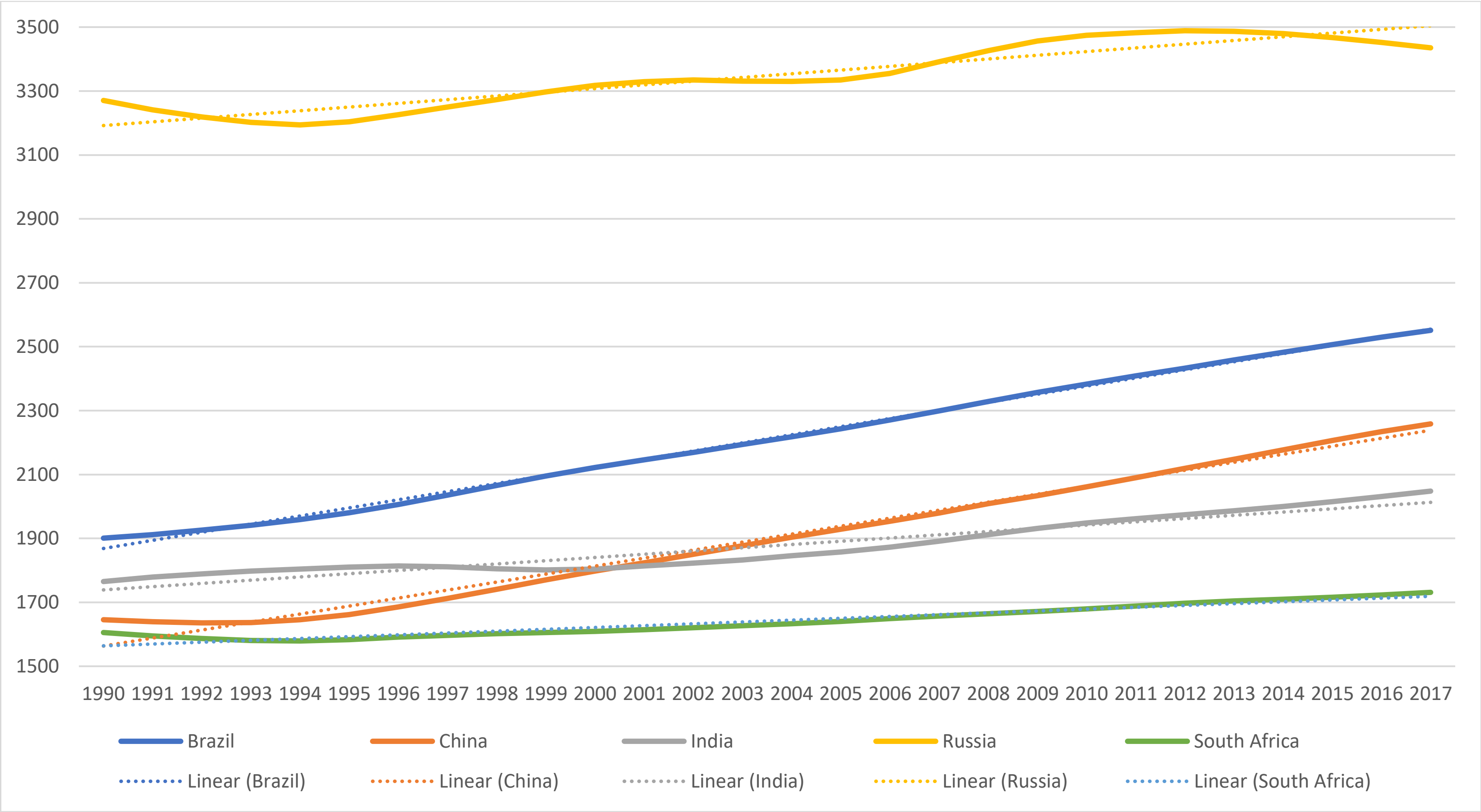

Supplementary Figure 5: Rehabilitation-sensitive YLD Rates (i.e. Years Lived with Disability per 100,000 inhabitants) specifically for the group of *musculoskeletal & pain* conditions across the five countries analyzed [1990-2017], and their *best fit* regression models (dotted lines).

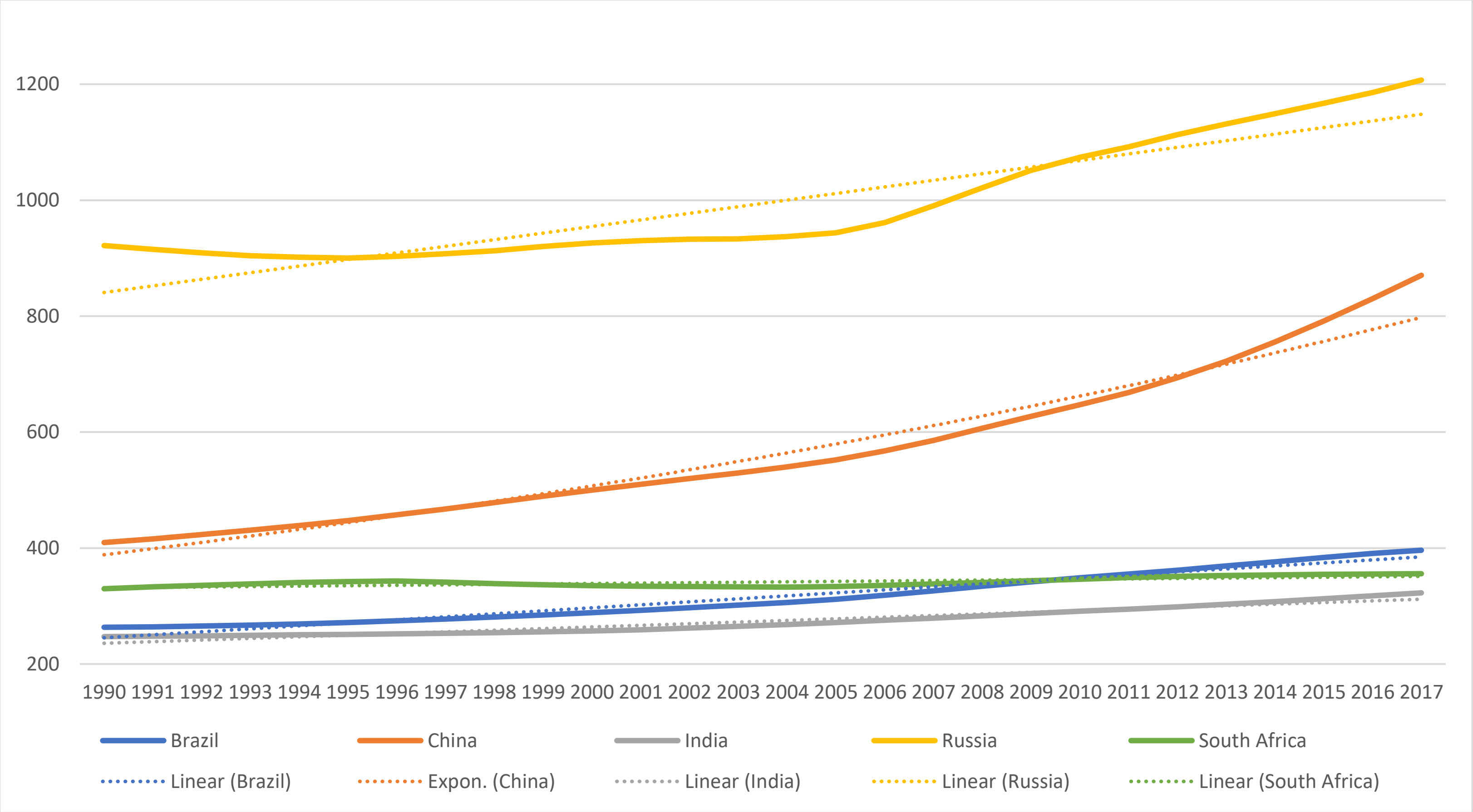

Supplementary Figure 6: Rehabilitation-sensitive YLD Rates (i.e. Years Lived with Disability per 100,000 inhabitants) specifically for the group of *neurological* conditions across the five countries analyzed [1990-2017], and their *best fit* regression models (dotted lines). Abbreviation: Expon. = Exponential.

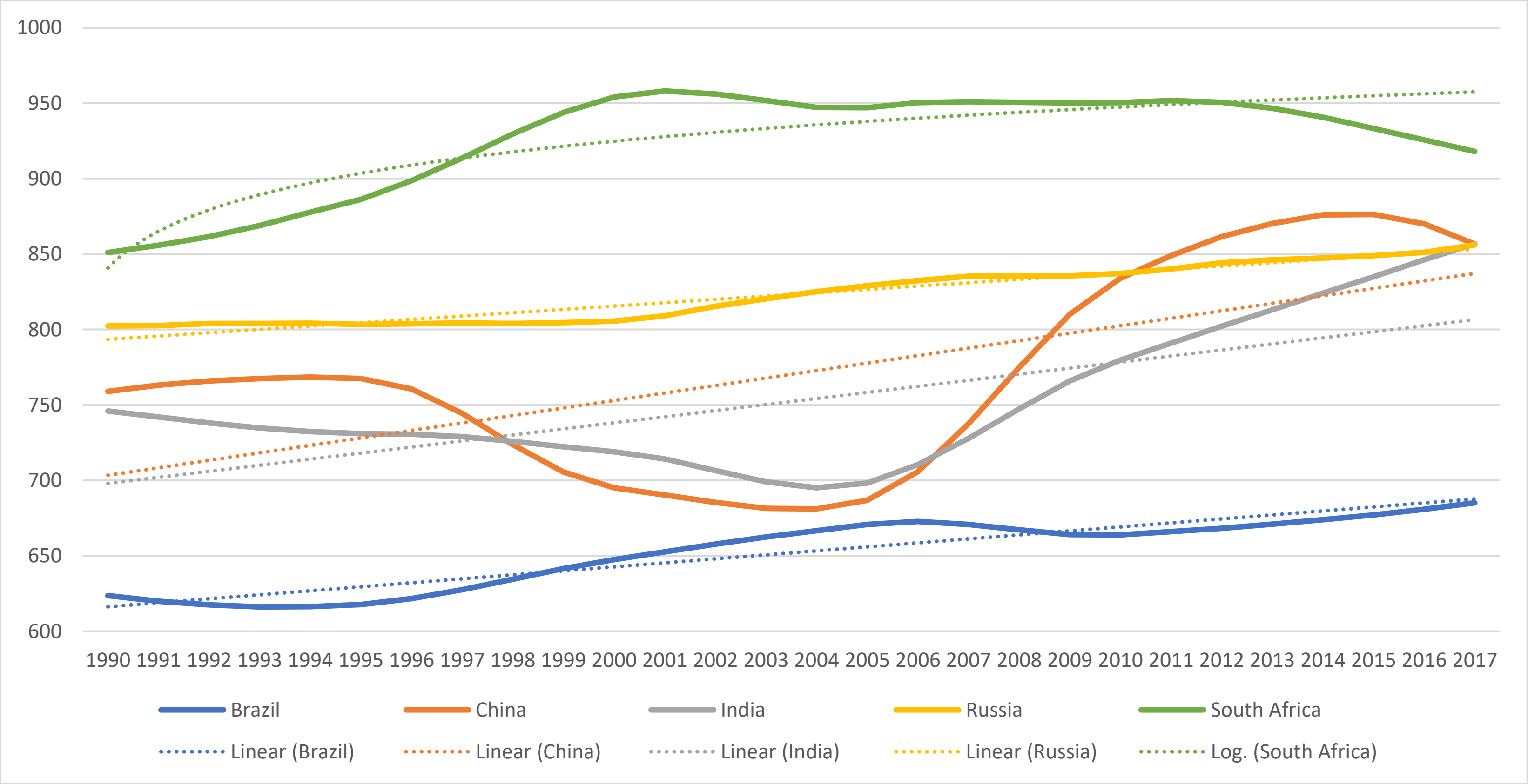

Supplementary Figure 7: Rehabilitation-sensitive YLD Rates (i.e. Years Lived with Disability per 100,000 inhabitants) specifically for the group of *cardiothoracic* conditions across the five countries analyzed [1990-2017], and their *best fit* regression models (dotted lines).

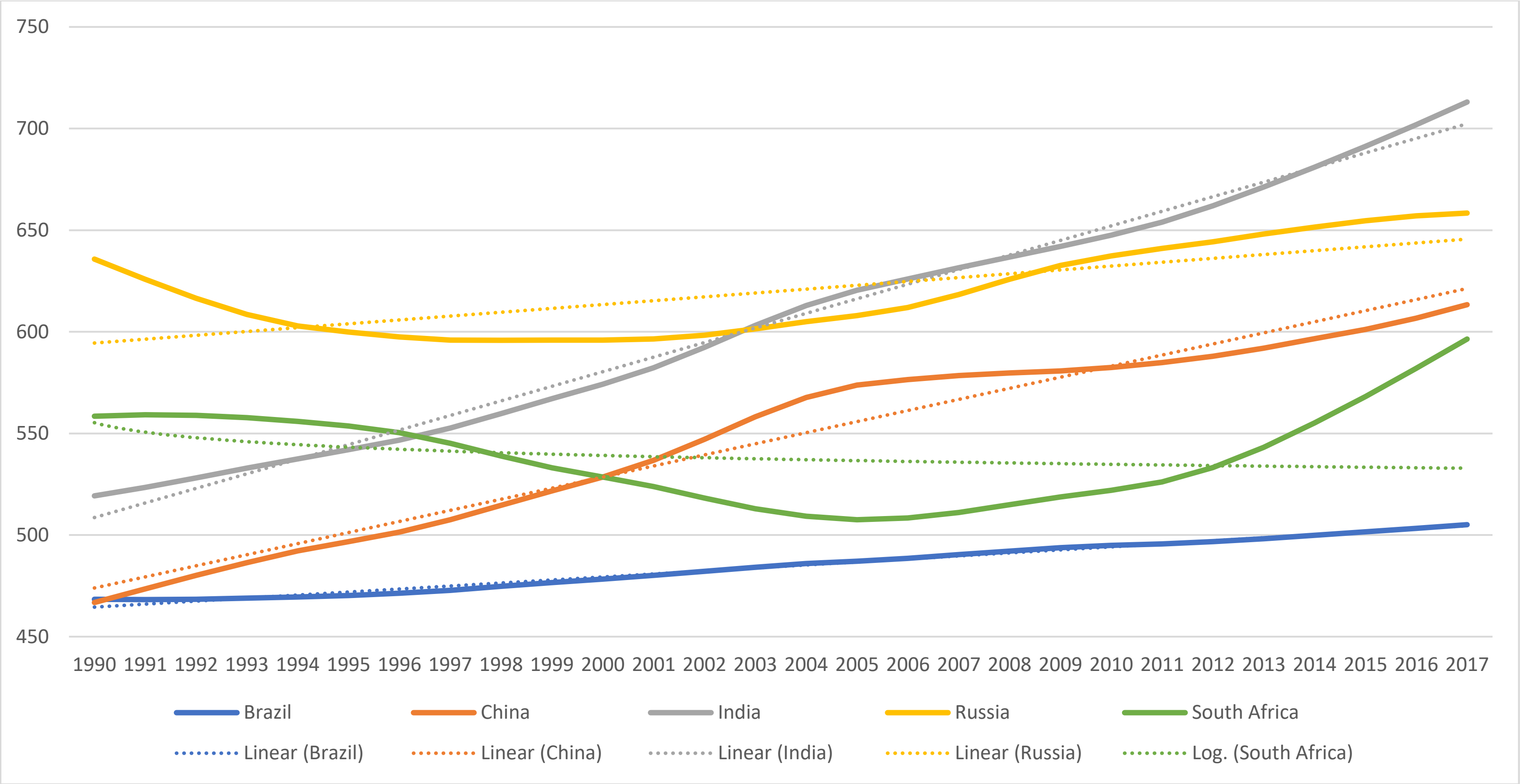

Supplementary Figure 8: Rehabilitation-sensitive YLD Rates (i.e. Years Lived with Disability per 100,000 inhabitants) specifically for the group of *pediatric* conditions across the five countries analyzed [1990-2017], and their *best fit* regression models (dotted lines). Abbreviation: Log. = Logarithmic.

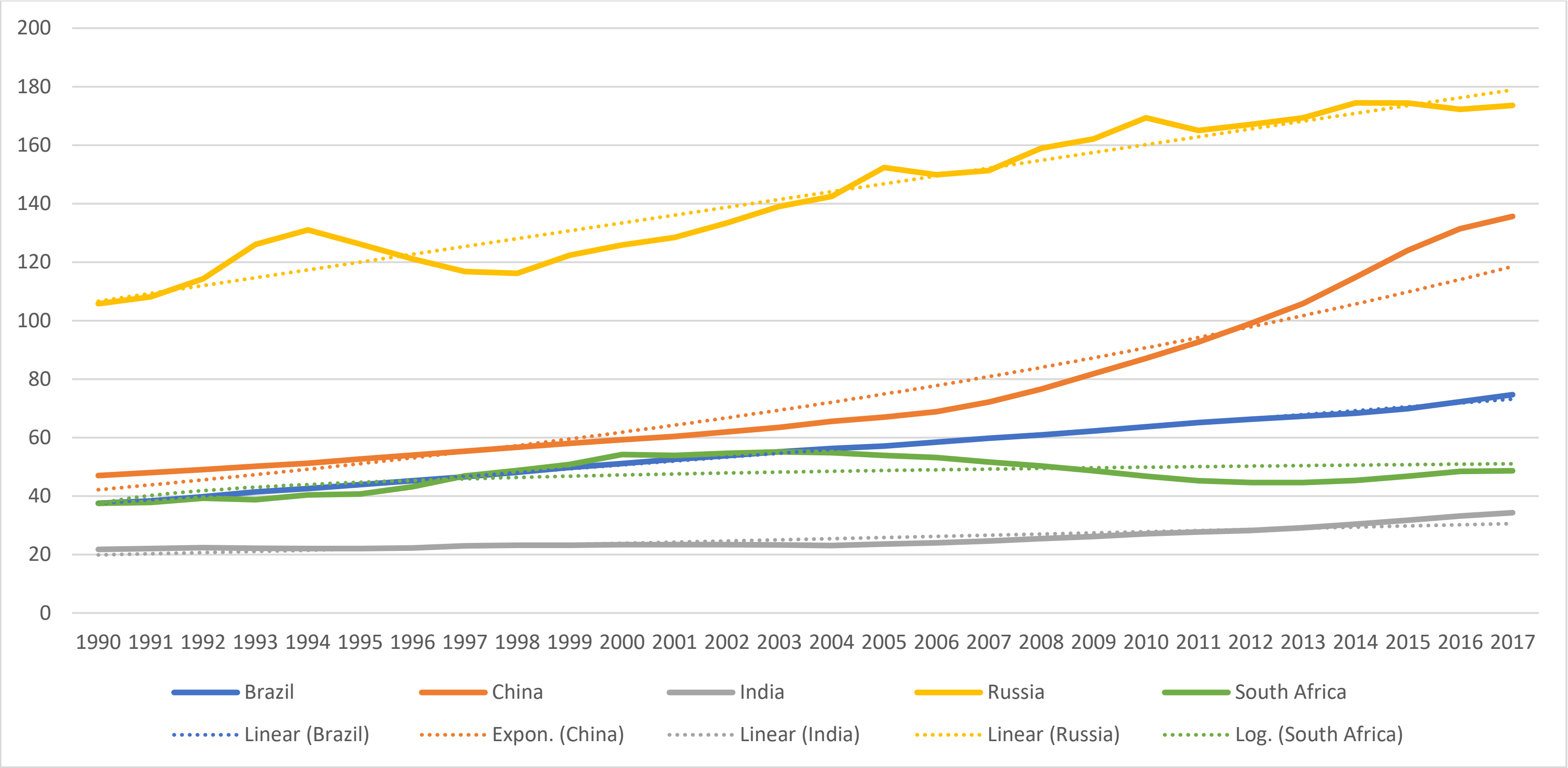

Supplementary Figure 9: Rehabilitation-sensitive YLD Rates (i.e. Years Lived with Disability per 100,000 inhabitants) specifically for the group of *neoplastic* conditions across the five countries analyzed [1990-2017], and their *best fit* regression models (dotted lines). Abbreviations: Expon. = Exponential; Log. = Logarithmic.

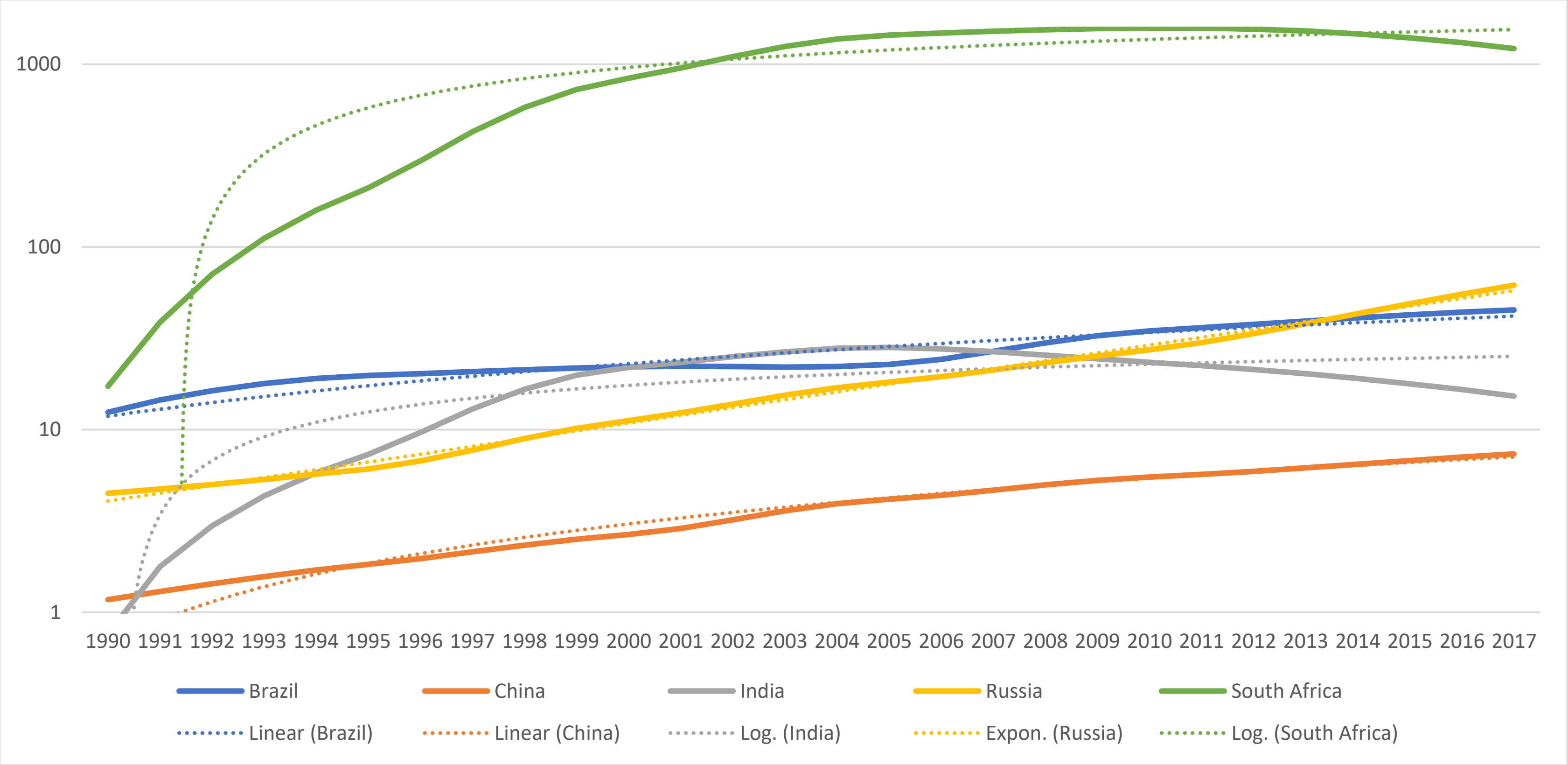

Supplementary Figure 9: Rehabilitation-sensitive YLD Rates (i.e. Years Lived with Disability per 100,000 inhabitants) specifically for the group of *HIV-related* conditions across the five countries analyzed [1990-2017], and their *best fit* regression models (dotted lines). The *Y* axis is at logarithmic scale. Abbreviations: Expon. = Exponential; Log. = Logarithmic.
